# Diagnostic and therapeutic dilemmas of asymptomatic intracranial AVMs: A case report and evidence-based review

**DOI:** 10.1016/j.radcr.2024.08.154

**Published:** 2024-09-26

**Authors:** Moustafa A. Mansour, Reem W. Malaeb, Hamdi Nabawi Mostafa, Mohamed Ibrahem Kamal, Basim Ayoub

**Affiliations:** a Department of Neurosurgery, Nasser Institute for Research and Treatment, Cairo, Egypt; b Department of Neurology and Neurologic Surgery, Faculty of Medicine, Al-Azhar University, Cairo, Egypt; c Department of Health Professions, Faculty of Health Sciences, American University of Beirut, Beirut, Lebanon; d Department of Neurosurgery, Misr University for Science and Technology, Giza, Egypt; e Department of Neurosurgery, Al Bank Al Ahly Hospital for Integrated Care, Cairo, Egypt; f Department of Neurosurgery, El- Sahel Teaching Hospital, Cairo, Egypt; g Department of Neurosurgery, Ismailia Medical Complex Hospital, Ismailia, Egypt; h Department of Neurosurgery, Faculty of Medicine, Cairo University, Cairo, Egypt; i Department of Neurosurgery, Kasr Al-Aini Hospital, Cairo, Egypt

**Keywords:** Arteriovenous malformations complications, Arteriovenous malformations diagnostic imaging, Arteriovenous malformations therapy, AVM, Brain blood supply, Brain diagnostic imaging, Intracranial arteriovenous malformations, Rupture, Intracranial hemorrhage, Cerebral hemorrhage etiology, Radiosurgery, Case report, Neuroradiology

## Abstract

First described by Virchow in the 19th century, intracranial arteriovenous malformations (AVMs) are complex, tangle-shaped vascular lesions with a number of associated neuroparenchymal, hemodynamic, and angio-architectural changes. However, the clinical description of extracranial AVMs dates back to the Ebers Papyrus (c. 1500 BC), with a still unknown definitive underlying etiology thus far. AVMs are rare lesions, with approximately 0.15% incidence and 0.001-0.5% prevalence, but of high importance as they tend to affect young patients who are frequently otherwise healthy. In the majority of cases, AVMs present as sudden intracranial hemorrhages that require immediate intervention, but incidentally-detected unruptured AVMs are only found in ∼15% of cases, leaving a confusing dilemma regarding the appropriate next step, particularly given the several therapeutic interventions available and clinical trials that were vulnerable to follow-up criticism. Herein, we present a case of an incidentally detected asymptomatic AVM in a 15-year-old boy via advanced imaging techniques that was initially misinterpreted as a post-traumatic subarachnoid hemorrhage on routine imaging studies. In providing a comprehensive overview of pathological classification schemes and the currently available diagnostic options for these silent dilemmatic AVMs, we highlight three management techniques: microsurgical resection, endovascular embolization, and stereotactic surgery, with the best option depends mostly on addressing lesion resection properly with minimal associated mortality and morbidity.

## Introduction

**Fig. 1:**
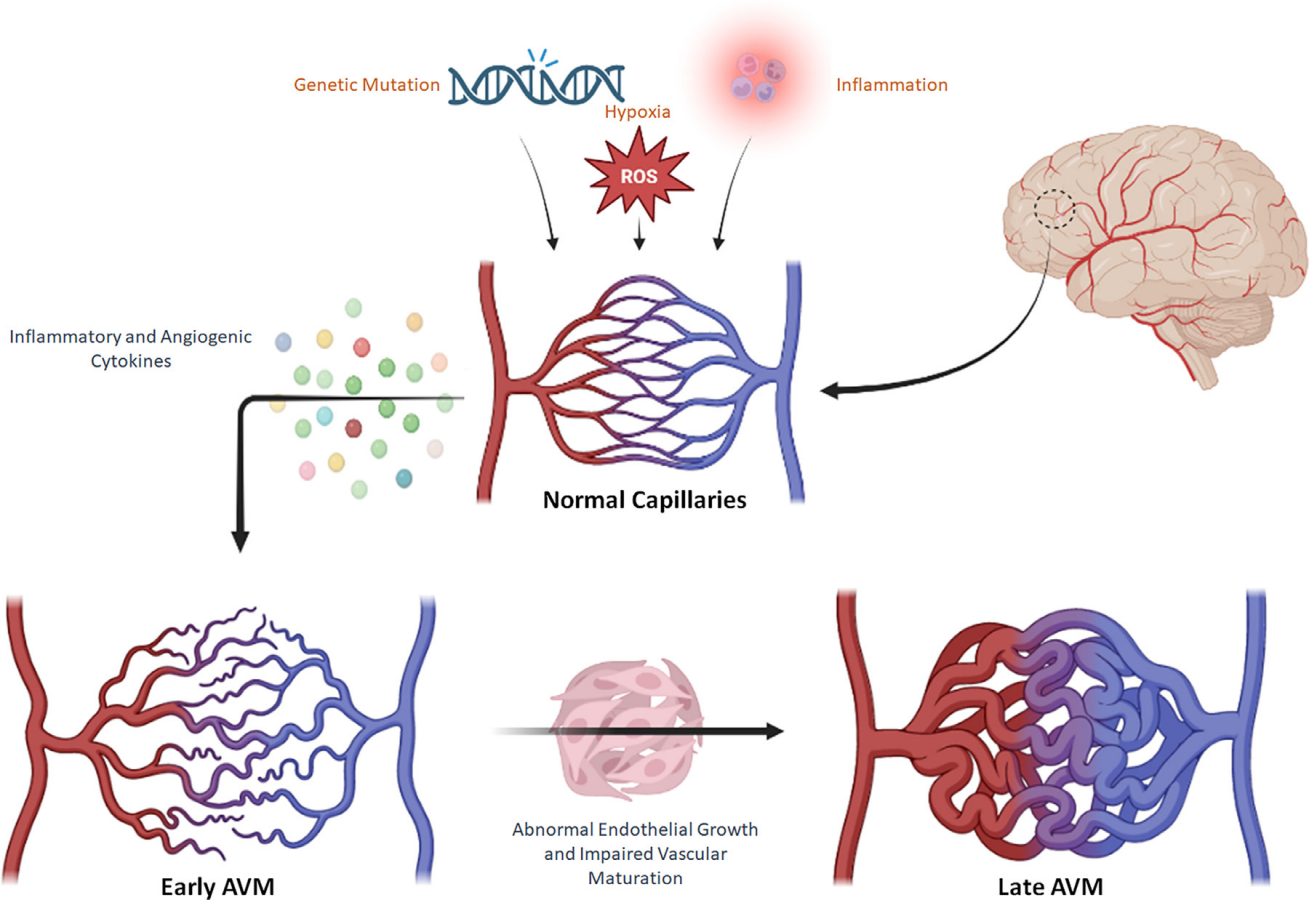
Schematic illustration of the postulated pathogenesis of brain arteriovenous malformations. (ROS: reactive oxygen species)

The fundamental abnormality in AVMs is one or more direct fistulous communications between afferent feeding artery/arteries and efferent draining vein(s) without an intervening capillary bed. Subsequently, the increased blood flow through these abnormal shunts causes the characteristic changes of vascular dilatation, tortuosity, and the formation of the characteristic nidus. AVMs are thought to develop during embryonic development, either via a retention of the primordial arteriovenous connections or an induced agenesis affecting the capillary system of the fetal circulation [1]. However, other theories postulated that AVMs might be due to a progressive hemodynamic abnormality rather than a static congenital deficit [2]. Specific genes, such as endoglin and ALK-1, have been reported to be in association with the development of AVMs in some cases [3]. However, there may be clear evidence of *de novo* formation and active growth postnatally, suggesting a more complex mechanism, involving environmental contributions (e.g., hypoxia) coupled with unregulated inflammatory response (Fig. 1).

AVMs typically present as sudden intracranial hemorrhages requiring an urgent intervention, while incidentally-detected unruptured AVMs can only be found in about 15% of cases, raising a confusing dilemma regarding the appropriate next step, particularly given the wide variety of therapeutic interventions and clinical trials that were not immune to follow-up criticism.

Herein, we report a case of a silent brain AVM that was incidentally detected in a teenager after being initially misdiagnosed as minor subarachnoid bleeding following mild head trauma, using specific magnetic vascular imaging as well as volume rendering techniques. We additionally review the currently available management options for these lesions and elaborate on how the management decision is favored between these management options.

## Case presentation

**Fig. 2:**
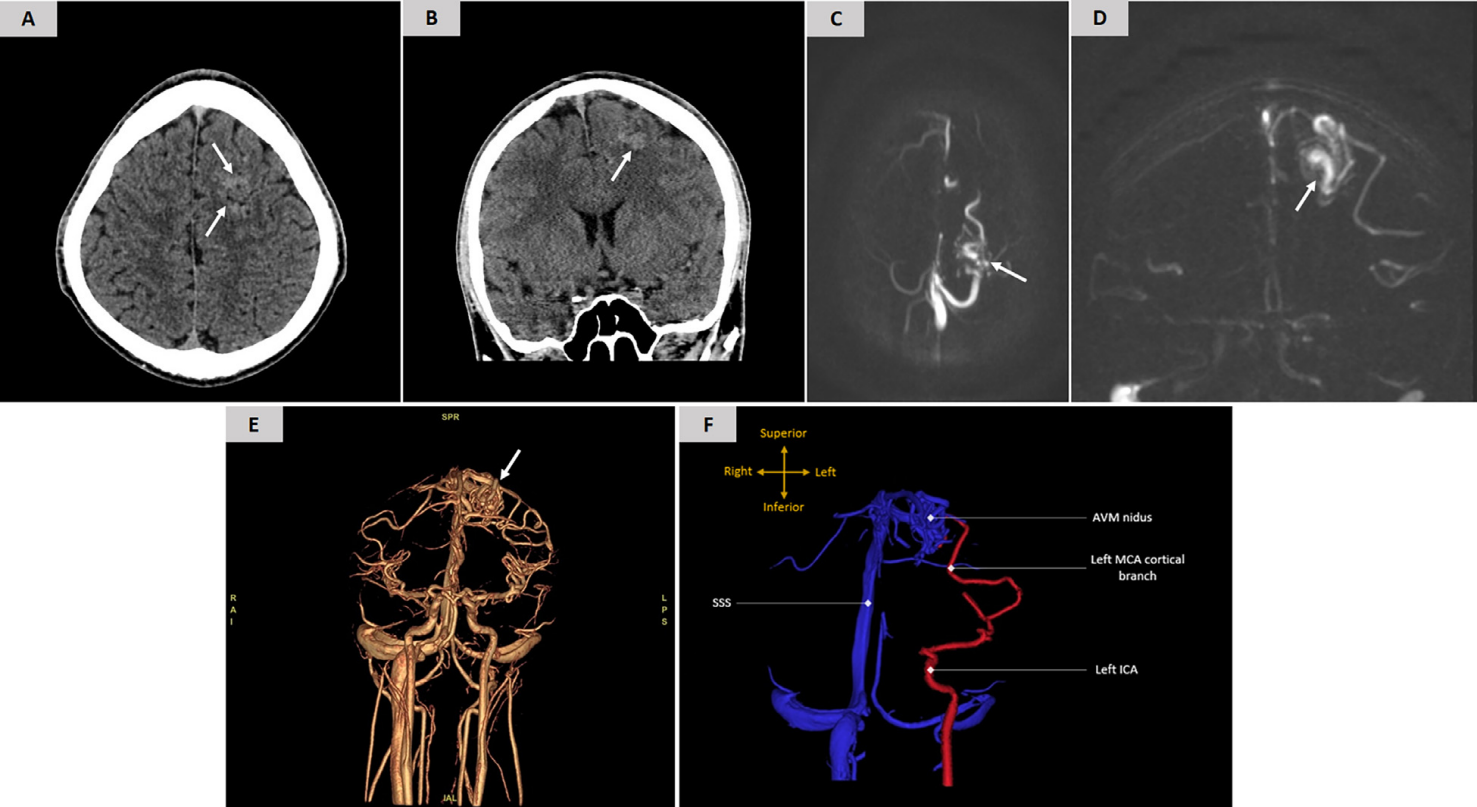
(A) Axial and (B) Coronal head CT images demonstrate a tuft of abnormal vessels located on the left superior frontal gyral surface (*arrows*). (C) Axial and (D) Coronal MR venogram images reveal a vascular nidus of approximately 2.0 × 2.0 cm involving the left superior surface of the frontal lobe (*arrows*). (E) VRT reconstruction image and (F) Annotated depiction of the arterial feeder, nidus, and venous drainage of the detected AVM lesion. (AVM, arteriovenous malformation, MCA, middle cerebral artery, SSS, superior sagittal sinus, ICA, internal carotid artery)

A previously healthy 15-year-old boy was brough by his mother to the emergency department (ED), after accidently falling down the stairs and mildly hitting the left side of his head. Subsequently, he had a mild dull-type headache localized to the same side. He denied any associated visual disturbance, photophobia, phonophobia, nausea, vomiting, or dizziness. He reported no previous incidents of head injury. Examination of his head, neck, and cranial nerves was unremarkable. Furthermore, strength, Joints' range of motion, sensation, gait, balance, coordination, and deep tendon reflexes were within normal. Additionally, the patient was fully oriented to month, date, day of the week, year, and time. He also was able to recall five words immediately and five minutes later, and a head CT trauma protocol was then performed. During the scan, the CT technician flagged a left frontal subarachnoid hemorrhage seen in the left superior frontal gyral surface, but upon our review of the images, we realized that it could be an abnormal vessel (Figs. 2A and B). Therefore, we opted to do a correlative magnetic resonance (MR) venogram to identify if that was anything more than a developmental venous anomaly. MR venogram revealed an arteriovenous malformation involving the left superior frontal lobe with a nidus of approximately 2.0 × 2.0 cm, with direct arterial feeders from cortical branches of the left middle cerebral artery and venous drainage into the superior sagittal sinus (Figs. 2C and D). Volume rendering technique (VRT) was used to display a 2D projection of the 3D data set (Figs. 2E and F) (Videos 1 and 2_supplementary materials), and the diagnosis of a cortical AVM was made. Based on the elements of size, venous drainage, arterial supply, and location of the lesion, as well as the patient's age and race, the patient's AVM lesion was granted a Spetzler-Martin (SM) score of 2, and R₂eD AVM score of 2 with a 32% risk of hemorrhagic presentation and a 90% cumulative AVM rupture risk. In view of these data and after discussing with the parents, the recommendation of the neurovascular multidisciplinary meeting was staged gamma knife radiosurgery, which was subsequently delivered with a good outcome in the form of progressive reduction in the size and flow rate of the AVM over a 6-month period. The patient was then scheduled for annual MRI scans to follow up on nidus obliteration.

## Discussion

### Pathological features

**Fig. 3:**
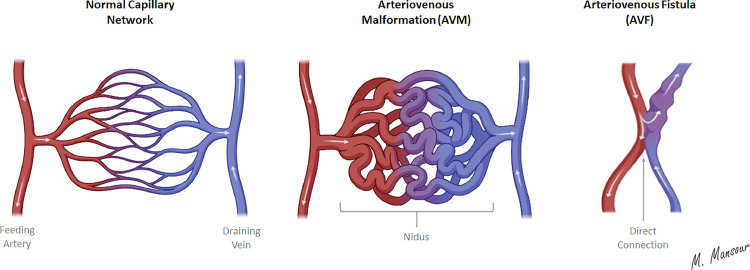
Schematic illustration highlights the pathological features of arteriovenous malformations and arteriovenous fistulas.

**Fig. 4:**
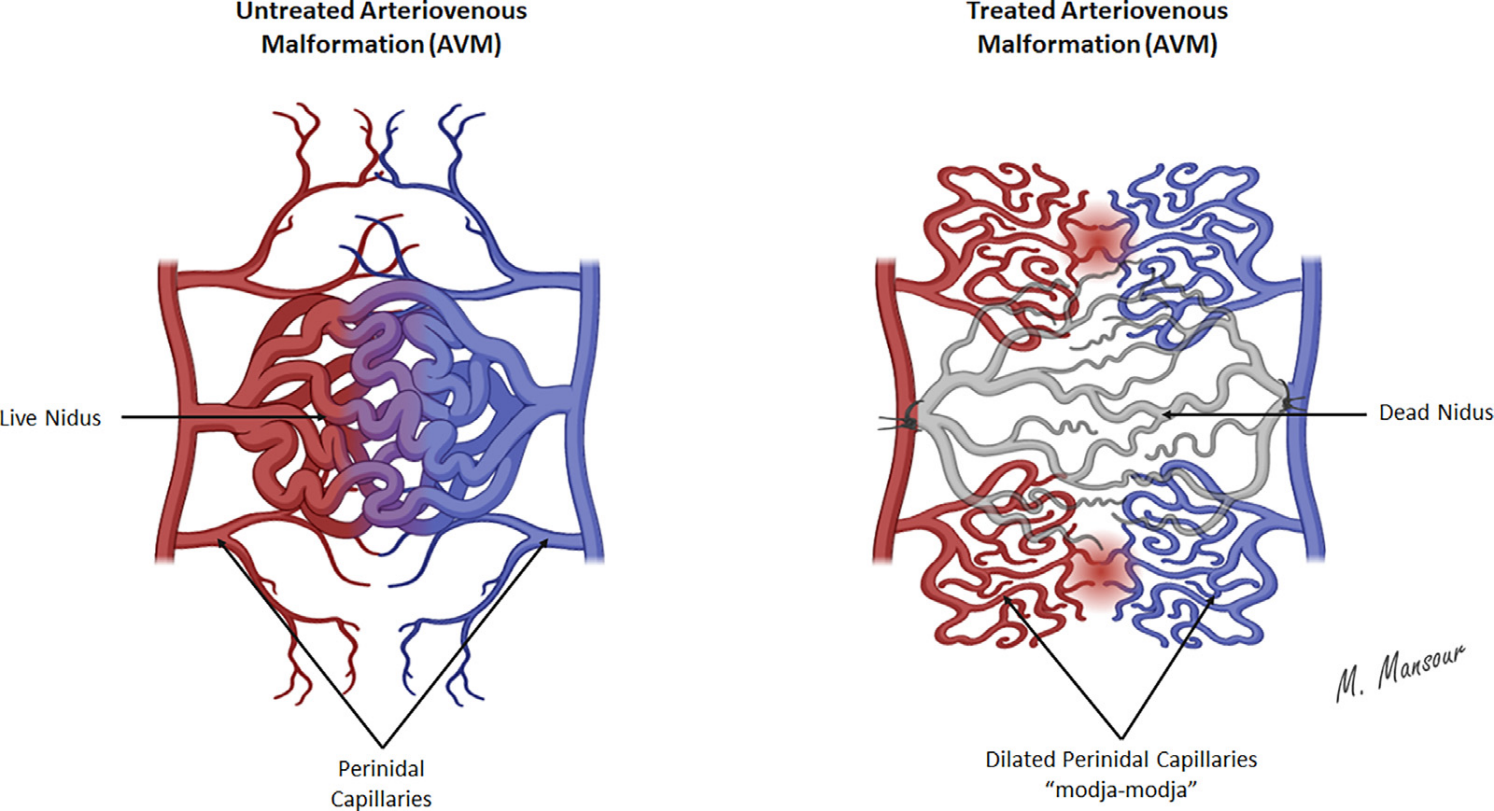
Schematic depiction of perinidal vessels associated with arteriovenous malformations with/without treatment.

AVM lesions typically consist of arterial feeders, draining veins, and nidus. The nidus, or the functional unit of the AVM, is a tangle-shaped vascular network that connects feeding arteries and draining veins without a functioning capillary bed. Another mimetic entity of AVMs, previously referred to as a subtype of AVMs, is the arteriovenous fistulas (AVFs). However, these AVFs lack the characteristic nidus (Fig. 3) [4]. Proximal to the AVM's nidus, the perinidal capillaries are located, forming potential vascular spaces that are assumed to get hemodynamically overloaded, referred to as modja-modja (Fig. 4), upon attempted nidal resection with a subsequent increase in the intravascular pressure, leading to rupture and bleeding [5,6]. Given the absence of intervening capillary beds in AVM lesions, the arterial feeders finally dilate due to the absence/low capillary-mediated resistance. Therefore, feeding arteries might exhibit high, turbulent blood flow, making them prone to developing arterial aneurysms. Venous aneurysms may also develop, given the increased venous pressure secondary to the direct communication(s) between these veins and the arterial feeders. In some cases, AVM lesions might contain scattered areas of dystrophic calcification, gliotic tissue, or minute hemorrhages at different stages, attributed to the resultant hemodynamic and angioarchitecture changes. Intracranial AVM lesions affect the supratentorial compartment in approximately 85% of cases with either superficial or deep locations, while infratentorial involvement comprises ∼15% of cases. Intracranial AVMs might present as solitary or multiple lesions, with the latter being the least predominant (2%-5%) and commonly detected in association with vascular syndromes, such as Osler–Weber–Rendu (also known as hereditary hemorrhagic telangiectasia) syndrome [7].

### Epidemiology and clinical presentation

AVMs are rare lesions, with approximately 0.15% incidence and 0.001%-0.5% prevalence, but of high importance as they tend to affect young patients who are generally otherwise healthy [8]. In a large study by Hernesniemi *et al.* [8], AVMs were mostly presented as intracranial hemorrhages (ICHs), accounting for approximately 2% of all strokes and 4% of intracranial hematomas, with up to 38% among 15-45 age group. Hemorrhage from a bleeding AVM is associated with a 5%-10% risk of death and a 30%-50% risk of permanent or disabling neurologic deficits [8,9]. Other AVMs-associated presentations may include seizures, that were estimated as the second most common AVM-associated presentation per a multicenter study of 1289 patients which reported that ∼30% of included patients presented with generalized seizures, while 10% presented with focal seizures [10]. In the same study, the incidence of patients presented with seizures in the absence of hemorrhages was between 17% and 40% [10]. The exact etiology of epileptogenesis in cerebral AVMs is still unclear. However, recent data suggested primary and secondary causes of epileptogenesis in AVM cases. Focal cerebral hypoperfusion due to a steal phenomenon caused by arteriovenous shunting has been suggested as the primary cause of epileptogenesis, while the secondary cause has been attributed to the presence of gliotic tissues within the AVM nidus. Less commonly, ipsilateral headaches and focal neurologic deficits can be found in 3%-10% of AVM patients, which are thought to be attributed to either a resultant mass effect or hemodynamic disturbances. It is worth mentioning that headaches were reported as the second most common presentation (seizures as the third) in a comparative study conducted by Zhao *et al.* [11] which included 2086 consecutive patients with intracranial AVMs.

### Diagnostic evaluation

Given that approximately 15% of AVM patients can be asymptomatic at the time of detection, AVMs are considered a clinically silent disease entity; however, this figure should increase with greater access to MRI scanning nowadays. AVMs may present as a hyperdense lesion on routine noncontrast CT imaging, mostly large-sized lesions, making them misinterpreted as focal hemorrhagic lesions, as in our case. On the T2-weighted MRI sequence, AVMs demonstrate flow voids tangles, representing the nidus, with/without areas of hemorrhages and calcifications. Functional MRI might be used in the preoperative assessment to determine the neighboring eloquent brain areas to avoid during resection or embolizations and reduce postoperative complications. CT and MR angiography can provide a better delineation of vascular anatomy, specifically with 3D vascular reconstruction. Yet, digital subtraction catheter angiography remains the gold standard, providing a comprehensive evaluation of venous outflow obstruction, associated aneurysms, feeding artery/arteries, and pattern of venous drainage. Rotational angiography with 3D reconstruction has been of paramount importance in AVM imaging, given its precise depiction of the angioarchitecture of AVM lesions, aiding impactfully in deciding the most appropriate next step ahead, such as the technical feasibility of microembolizations and revealing any hidden AVM-associated compartments, hence the most appropriate surgical resection plan (if applicable). Furthermore, it aids in the precise measurement of parameters required for staging and classifications, such as size, location, and pattern of venous drainage, hence defining the severity and rupture risks of the lesions.

### Classification systems

AVMs-related dilemmas extend beyond diagnosis and management to include establishing a well-recognized classification scheme for AVM lesions as per most other neurologic disorders. Over the preceding years, several scales for AVMs classification have emerged to predict the associated morbidities and mortalities and assess the risks of different interventions. However, the Spetzler-Martin (SM) scale, formulated in 1986, remains the most quoted classification system [12]. The SM scale depends on three AVM-related parameters (size, venous drainage, and location eloquence) to generate a score out of five, with higher scores translating to higher SM grades (I-V), hence a greater risk of morbidity and mortality (Table 1). In 2011, a consolidation of the SM scale came out by Spetzler and Ponce, transforming the 5-tiered standard scale into a 3-tiered system by combining grades I and II into class A and grades IV and V into classes C while referring to grade III as class B [13]. The reason that drove the authors to suggest that transformation relied on enhancing the statistical power for comparative studies in such a rare pathological entity based on similar surgical results in the unified SM grades. While the SM scale is the standard grading system in the management of AVM lesions, some authors have proposed an additional 5-tiered system to complement the standard SM scale, taking into account specific parameters: age, hemorrhagic presentation, and lesion compactness (Table 2), to yield a final 10-point grading scale, in conjunction with the standard SM grading system [14], that has demonstrated a higher accuracy with preoperative risk prediction and extrapolation of neurologic outcomes [15]. Although the SM grading scale is the most widely adopted classification system for AVM lesions, another system known as the Toronto scale has demonstrated superior extrapolation over the standard SM system even when combined with its supplementary scale. In the 9-point Toronto scale, the predictive variable's influence depends on its relatively estimated weight, with eloquent location maximized by the score of “4”, uncompacted diffuse nidus by the score of “3”, and deep venous drainage by the score of “2”, in order to estimate the probability of neurologic deficits accordingly (Table 3) [16]. Due to the diversity and continuous developments concerning the management plans of intracranial AVM lesions and prioritizing stereotactic radiosurgery as a management option for unruptured AVMs in several centers, a specified grading system for radiosurgery, named Pollock-Flickinger AVM scale (*modified from the original BRAS system*), has emerged, which takes into account the AVM location and volume, besides the patient age, with multiple encouraging validations irrespective of the radiosurgery technique used [17,18]. The 5-point Buffalo scoring system is the most recent grading scale in managing intracranial AVMs, representing a new tool with potential superior advantages in guiding endovascular embolization of intracranial AVM lesions, accounting for the number of arterial pedicles, the diameter of arterial pedicles, and the presence or absence of neighboring eloquent brain areas (Table 4) [19]. However, it requires additional external validation since it relied on the retrospective analysis to compare the predictive accuracy to the standard SM system. Furthermore, only 50 patients were included in that retrospective analysis, giving it a relatively lower statistical power when compared to standard grading systems.

**Table 1:** Spetzler-Martin grading system for cerebral arteriovenous malformations.

Parameter		Points
Size (cm)	<3	1
	3-6	2
	>6	3
Venous Drainage	Superficial	0
	Deep	1
Eloquence	No	0
	Yes	1
Total		1-5

**Table 2:** Lawton supplementary scale for cerebral arteriovenous malformations.

Parameter		Points
Age (y)	<20	1
	20-40	2
	>40	3
Hemorrhagic Presentation	Yes	0
	No	1
Compactness	Yes	0
	No	1
Total		1-5

**Table 3:** The Toronto scale for the assessment of surgery-related risks for cerebral arteriovenous malformations.

Risk	Points	Probability of neurologic deficit(s) with surgery (%)
Low	0-2	1.8
Moderate	3-5	17.4
High	6-7	31.6
Very High	>7	52.9

**Table 4:** Buffalo grading system for endovascular treatment of brain arteriovenous malformations.

Parameter		Points
Number of arterial pedicles	1-2	1
	3-4	2
	≥5	3
Diameter of arterial pedicles (mm)	>1	0
	≤1	1
Eloquence	No	0
	Yes	1
Total		1-5

### Management techniques

Unruptured AVMs tend to cause bleeding at a lower rate than those that have ruptured. Furthermore, unruptured AVMs may have higher treatment-related morbidity than ruptured AVMs, besides the different management modalities available, accounting for the main focus of the debate regarding the most appropriate management plans [20,21]. Management of AVMs utilizes 3 management techniques, alone or in combination. These include microsurgical resection, endovascular embolization, and stereotactic radiosurgery. The manner in which these are applied takes into account certain patient-related factors such as the age, presenting symptoms, and the presence/absence of comorbidities, as well as certain AVM-associated factors such as the nidus size and location, in addition to the available expertise. With the advent of endovascular obliteration and gamma knife radiotherapy, neurosurgical intervention is no longer the approach of choice for unruptured intracranial AVMs in most centers. However, for some neurosurgeons, microsurgery is considered the favored first-line approach for unruptured AVMs with low SM grades due to the lower rates of associated morbidity and mortality [10,11,22].

Stereotactic radiosurgery, via proton beam, gamma knife, linear accelerator (LINAC), or CyberKnife, is typically reserved for compact, small-sized lesions, because such small-sized lesions do not typically harbor normal brain tissue that might get inversely affected by the radiation beams and subsequently convert into a gliotic epileptogenic tissue. It is currently the preferred treatment strategy for the majority of patients unless there are good clinical reasons to prefer surgery (e.g., treatment of the AVM during removal of a hematoma or patient preference after careful counseling). After treatment with stereotactic radiosurgery, which may take up to 4 years to obliterate the nidus, there is a reported risk of hemorrhages that have been estimated at 4.8%-7.9% per year for the first 2 years, then 2.2%-5% in the third, fourth, and fifth years. Notably, if the nidus is successfully obliterated, then the risk of future hemorrhages will be near zero. However, Maruyama *et al.* [23] reported that 6 out of 250 patients bled after a confirmed “cure” of their AVMs. In the optimal case, with a small-sized compact nidus in a noneloquent location, complete obliteration can be achieved in at least 90% of cases; however, this decreases significantly for larger AVMs that usually require relatively lower doses of radiation to avoid any radionecrosis-induced hemorrhage. The underlying therapeutic mechanism of radiosurgery in AVMs is attributed to radiation-induced endothelial injury that leads to vascular smooth muscle hyperproliferation with subsequent vascular occlusion. Ideally, follow-up after radiosurgery would involve annually-conducted MR imaging scans, which are used as a preliminary indication of nidus obliteration, and will often require 2-3 years but may take up to 4 years from starting therapy. A meta-analysis of sixty-nine cohorts by Beijnum *et al.* [24] found that ∼40% of AVMs treated with radiosurgery reach a full obliteration within approximately 24 months. During follow-up after radiosurgery, once there are no obvious flow voids in the area of treatment on MR imaging, a digital subtraction angiography should be indicated to confirm obliteration. Retreatment with radiosurgery is possible and would ideally be recommended if obliteration has not occurred within 4 years.

Endovascular embolization has been reported to provide good adjuvant benefits if used before microsurgery. However, as a primary method, it offers relatively low cure rates (<20%) with a risk of subsequent recanalization. Therefore, endovascular approaches are generally preserved for certain circumstances such as cases where AVMs are located in deep brain areas (e.g., thalamus, or the basal ganglia) with a few numbers of targetable feeding arteries. However, the use of new embolic agents, such as Onyx® (ethylene-vinyl-alcohol copolymer), has been reported to hold a promise for this technique, with cure rates of up to 50%. In large lesions, staged embolization followed by radiosurgery has been used with 2 major benefits; eradicating any AVM-associated aneurysms or fistulas, and shrinking the lesion so a higher radiation fraction can be subsequently delivered.

A staged multifaceted approach is typically recommended on a case-by-case basis, particularly with complex AVM lesions or after treatment failure. The staged multifaceted approach combines the advantages of individual treatments and may be the safest approach for some AVMs. However, it does result in an additive risk; therefore, careful consideration should be undertaken [24]. It is also worth mentioning that the current best evidence in relation to the treatment of unruptured brain AVMs is from the ARUBA trial, in which the authors of the clinical trial concluded that medical management was superior to interventional therapy [25]. However, several authors provided appropriate critiques of the study and its findings, citing design flaws, lack of standardization of the treatment arm, besides inadequate study details. To address the design flaws of ARUBA and evaluate the extent of efficacy and outcomes of the staged multifaceted management of AVM lesions, a new study called TOBAS was launched about 1 year after the primary completion of the ARUBA study. However, the inclusion criteria of TOBAS differ from ARUBA's as it involves ruptured and unruptured AVMs. The TOBAS results are supposed to yield significantly to the management guidelines of ruptured and unruptured AVMs. However, this will depend on the recruited number of appropriate patients. The trial might require up to 2000 participants in multiple centers (∼30) for a 10-year prospective follow-up to the estimated endpoint. However, the process seems to take longer than expected, with an estimated primary completion date of January 2035 [26].

### Clinical implications for health managers and policymakers

The ultimate decision for managing unruptured AVMs has to be taken in consultation with the patient, addressing the goal of achieving lesion resection or obliteration and symptom resolution with minimum associated morbidity and mortality. Management interventions are not risk-free. However, available expertise, besides the witnessed technical and technological advances, has been improving outcomes following the different management options. Therefore, the treatment of AVMs, irrespective of the individual case risk, should ideally be managed at specialized centers equipped with comprehensive expertise and multidisciplinary teams, ensuring the best possible outcomes.

### Conclusion and future direction

Intracranial AVMs are rare vascular lesions but with paramount clinical implications in respect of associated morbidity and mortality. Therefore, a comprehensive understanding of their pathogenesis, epidemiology, and typical and atypical presentation, on top of their natural history, is essential when formulating the most appropriate management option among other several management modalities currently available. Cerebrovascular imaging, either via MRI or catheter angiography, plays a paramount role in choosing the most suitable management option to maximize the rates of AVM obliteration and minimize the related morbidities. Despite the evidence-based results of the ARUBA study, interventional techniques are likely still warranted to remain the management option of choice for patients with unruptured low-grade AVM lesions. However, further well-established, high-powered, prospective studies for appropriately selected patients with unruptured AVM lesions are still warranted to affirm the beneficial outcomes of interventional methods over conservative treatments.

## Patient consent

The authors declare that they have obtained consent from the patient.
